# Immune Checkpoint Inhibitor-Induced Acute Pancreatitis and Colitis

**DOI:** 10.7759/cureus.8613

**Published:** 2020-06-14

**Authors:** Andrea Pagan, Yadis M Arroyo-Martinez, Ankita Tandon, Carlos Bertran-Rodriguez, Jeffrey Gill

**Affiliations:** 1 Internal Medicine, University of South Florida Morsani College of Medicine, Tampa, USA; 2 Internal Medicine, University of South Florida, Tampa, USA; 3 Gastroenterology, James A. Haley Veterans Affairs Hospital, Tampa, USA

**Keywords:** immune checkpoint inhibitors, pancreatitis, autoimmune colitis, diarrhea

## Abstract

Given the promising response of immune checkpoint inhibitors (ICPIs) in treating advanced malignancies, their use in clinical practice is on the rise. ICPIs are associated with a wide spectrum of immune-related adverse events (irAEs). The reported side effects of therapy can be severe enough to require interruption or withdrawal. We are presenting a case of a checkpoint inhibitor-induced acute pancreatitis and colitis, treated with high-dose steroids. This case highlights the need for all physicians to be aware of the different presentations of irAEs from checkpoint inhibitors to provide the correct diagnosis and management.

## Introduction

Immune checkpoint inhibitors (ICPIs) are an effective treatment for advanced malignancies. They work by releasing the brake that has been placed on the immune system, allowing the patients’ immune system to attack cancer cells, but also certain healthy tissues [1-3]. The primary targets for checkpoint inhibition include programmed cell death receptor 1 (PD1), programmed cell death ligand 1 (PD-L1), and cytotoxic T-lymphocyte-associated antigen 4 (CTLA-4). Antibodies inhibiting PD-1 (pembrolizumab, nivolumab) and PD-L1 (atezolizumab, avelumab, durvalumab) have been approved for treatment in several types of cancer, including melanoma of the skin, non-small cell lung cancer, kidney cancer, bladder cancer, head and neck cancers, and Hodgkin lymphoma [1-3]. As their use increases, their side effects have become more prevalent. These adverse events can be severe enough to require interruption or withdrawal of immune checkpoint blockade therapy [4]. The most common side effects involve skin rashes (46%-62%), autoimmune colitis (22%-48%), autoimmune hepatitis (7%-33%), endocrinopathies (thyroiditis, hypophysitis, adrenalitis, and diabetes mellitus; 12%-34%) [5]. We present a case of a checkpoint inhibitor-induced acute pancreatitis and colitis.

The abstract of this article has been presented at the American College of Gastroenterology Conference in October 2019 [6].

## Case presentation

**Figure 1:**
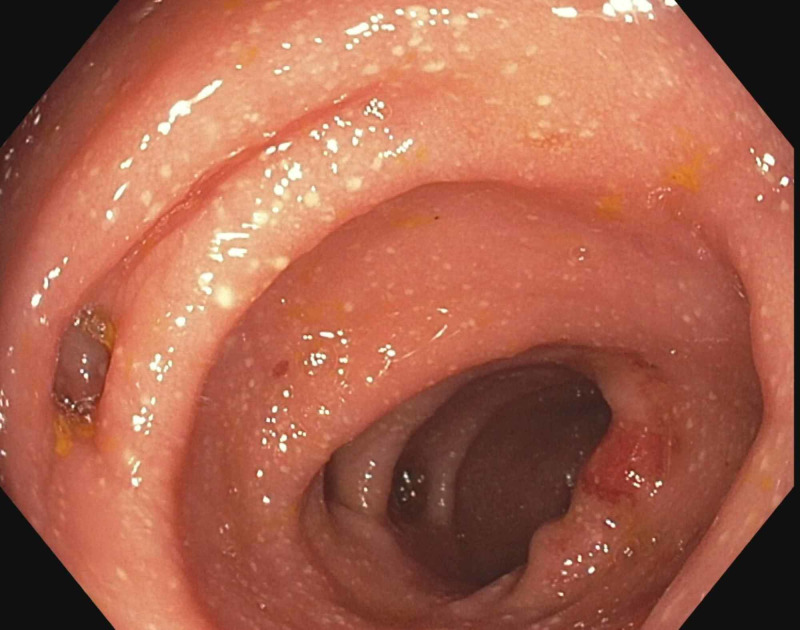
Colitis in the sigmoid colon and diverticulosis.

A 76-year-old male with medical comorbidities pertinent for controlled hypertension, and stage IV oligometastatic clear cell renal carcinoma that was being treated with pembrolizumab, presented to the emergency department (ED) with two days of abdominal pain, nausea, and vomiting. On physical examination, he was tachycardic, normotensive, and afebrile, with epigastric tenderness to deep palpation. Laboratory work revealed elevated creatinine of 1.5 mg/dL (0.84-1.21 mg/dL) and lipase of 436 U/L (0-160 U/L). Abdominal computed tomography showed edematous pancreas with loss of pancreatic lobulation. Other causes of pancreatitis were ruled out. The patient was diagnosed with autoimmune-mediated acute pancreatitis and was treated with high-dose steroids. Infusions of pembrolizumab were held until the steroid taper was over. Once infusions were restored, he began having non-bloody diarrhea, up to six stools per day. Physical examination and laboratory data were normal on the second presentation. His infectious and inflammatory workup for diarrhea was negative. He underwent a colonoscopy with endoscopic findings showing colitis in the sigmoid colon (Figure 1).

**Figure 2:**
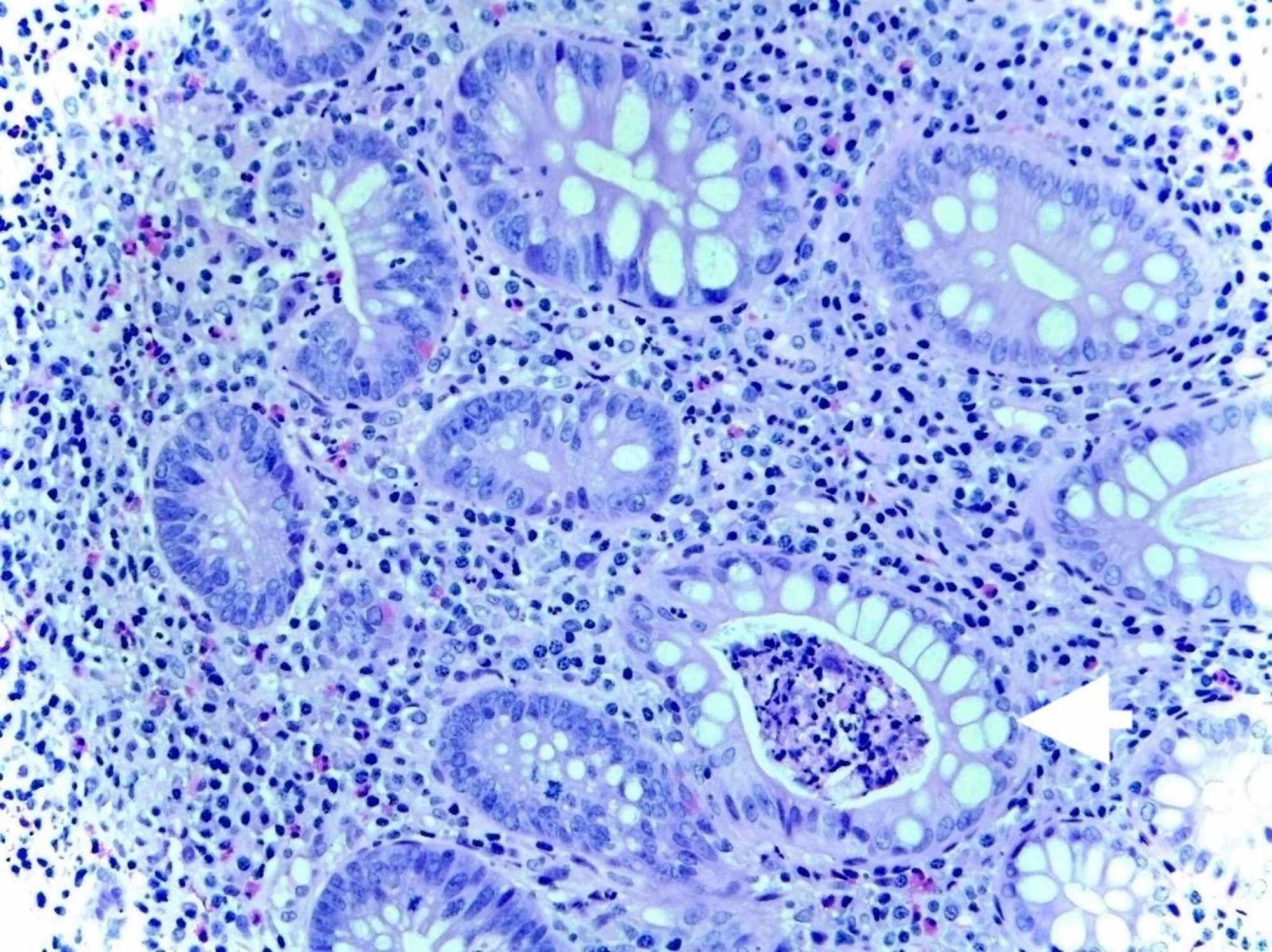
Active colitis with cryptitis and crypt abscesses (arrow) and chronic architectural alterations.

Random biopsies were obtained. Histological examination on the sigmoid colon showed chronic active colitis with crypt abscesses diagnosing the patient with checkpoint inhibitor-induced colitis (Figure 2).

He was restarted on high-dose steroids with improvement of his symptoms. Immunotherapy was placed on hold indefinitely.

## Discussion

Gastrointestinal toxicities are one of the most common causes of immune-related adverse effects of checkpoint blockade. Presentations include diarrhea and hepatotoxicity resulting in transaminitis and acute pancreatitis with the lowest incidence. Most immune-related adverse events appear within one to two months after the start of the checkpoint inhibitor [7]. Here we discuss the details of grading and managing ICPI-induced diarrhea as a reference for clinicians. Immune-mediated colitis and diarrhea is graded depending on the presentation. Grade 1 is defined as less than four stools per day above baseline. Grade 2 is defined as four to six stools per day and/or abdominal pain, mucus, or blood in the stool. Grade 3 is defined as seven or more stools per day and/or the presence of peritoneal signs with ileus and fever consistent with bowel perforation. Grade 4 consists of life-threatening consequences, such as hemodynamic collapse, perforation, ischemia, necrosis, bleeding, and toxic megacolon. Grade 5 consists of death [8]. Diagnosis is made by clinical history, laboratory workup, radiological imaging, sigmoidoscopy/colonoscopy, and histologic findings. Laboratory workup should be performed first to rule out any other cause of diarrhea or colitis, such as infectious. Abdominal computed tomography is useful to evaluate for obstruction, toxic megacolon, and inflamed areas due to ICPIs. Endoscopic and pathological findings are necessary to confirm ICPI-induced colitis and to rule out other causes of colitis, such as infectious, ischemic, other drug-induced, or diverticular colitis [9]. Endoscopic findings of immune-related diarrhea and colitis often show exudates, loss of vascular pattern, granular or edematous mucosa, patchy or diffuse erythema, aphtha, and ulcerations. The pathological evaluations most commonly show findings of active colitis, such as expansion of the lamina propria by lymphoplasmacytic infiltrate and later progressing to an increase in intraepithelial neutrophils and neutrophilic crypt abscess [9,10]. Treatment for colitis and diarrhea will depend on the grading. For grade 1, treatment consists of changes in diet, adding anti-motility agents, and continuation of ICPI therapy, once no infectious causes are identified. For grade 2, if immune-mediated colitis is identified on pathology and infectious workup is negative, treatment will consist of high-dose systemic steroids, 1-2 mg/kg/day, followed by a taper off corticosteroid therapy and ICPI treatment must be withheld until symptoms resolve [11]. For grade 3-4, hospital admission is recommended, and treatment includes intravenous high-dose corticosteroids, followed by a taper. Checkpoint inhibitor therapy must be discontinued permanently [12]. Early recognition of these toxicities is crucial in minimizing the impact of these complications on planned antineoplastic therapy.

## Conclusions

Gastrointestinal toxicities are among the leading causes of immune-related adverse effects of checkpoint inhibitors. Patients on treatment with ICPIs presenting with diarrhea and abdominal pain, ICPI-induced diarrhea, and colitis should be high on the differential. Other causes of diarrhea should be ruled out as part of the workup and endoscopic evaluation is usually required for it. Treatment for ICPI-induced diarrhea and colitis includes high-dose steroids, and it depends on the grade discontinuation of the offending agent. In order to provide the correct diagnosis and management, physicians should be aware of the different presentations of the side effects of the ICPIs.
